# Guilty by gown: reconsidering contact precautions for COVID-19

**DOI:** 10.1017/ash.2025.10055

**Published:** 2025-06-26

**Authors:** David Zamora Diaz

**Affiliations:** Internal Medicine Resident, Loyola University Health System - MacNeal Hospital, Berwyn, IL, USA

**To the Editor**

As a trainee, I often find myself donning gowns and gloves dozens of times per day when caring for hospitalized patients with confirmed or suspected COVID-19 infection. Despite our improved understanding of the virus transmission and the protection offered by respiratory Personal protective equipment (PPE), contact precautions remain the standard. With each plastic gown I tie on, sometimes the seventh visit to the same patient in a single day, an increasing uneasiness creeps in.

That guilt arises from multiple sources. First, the discomfort and stigma that patients experience while under contact precautions. Second, the substantial amount of plastic waste generated daily. Third, the cost burden placed on hospital systems. I frequently raise these concerns with attending physicians, questioning whether this routine use of gowns and gloves is still warranted. The answer is often the same: “We do it because it’s what the CDC recommends.”

The Centers for Disease Control and Prevention (CDC) continues to recommend both airborne and contact precautions for hospitalized patients with COVID-19, requiring healthcare personnel to wear gowns and gloves in addition to respirators and eye protection.^1^ While these recommendations were appropriate during the early, uncertain days of the pandemic, they may no longer be fully supported by current evidence.

We now understand that SARS-CoV-2 is primarily spread through aerosol transmission, not through contact with contaminated surfaces.^2^ Fomite transmission, once a major concern, now appears to play a minimal role in real-world settings. While hand hygiene remains crucial, the additional benefit of gowns and gloves for COVID-19 patients in routine clinical scenarios is less clear.

Others have raised similar questions about the continued role of contact precautions for COVID-19. Rodriguez-Nava et al. recently called for a reassessment of routine gown and glove use in healthcare settings, citing minimal evidence of benefit and increasing concern about harms.^3^ Rabin et al. also urged the infectious diseases community to reconsider this practice, arguing that outdated precautions could undermine both patient-centered care and public trust.^4^ These concerns are consistent with the growing sentiment among frontline providers: our infection control strategies must evolve with the evidence.

The potential harms of routine contact precautions are well-documented. Patients in isolation report greater feelings of loneliness, stigma, and dissatisfaction with care. Providers may reduce time spent at the bedside, resulting in fewer clinical reassessments and lower quality of care.^5^ Contact precautions have been associated with delays in imaging, lab tests, and specialist consultations, delays that may directly impact outcomes.

Beyond patient care, the environmental impact appears to be significant. Gowns and gloves are made of single-use plastics. The healthcare sector is already a major contributor to plastic pollution and carbon emissions. Continued overuse of unnecessary PPE only exacerbates this crisis.

Recent research has linked microplastic accumulation to adverse health outcomes. Microplastics have now been detected in human blood, placental tissue, and most alarmingly, brain tissue.^6^ A study by Nihart, et al. demonstrated the presence of synthetic particles in post-mortem brain specimens, raising concerns about neurotoxicity and systemic inflammation. Another investigation identified nanoplastics embedded in atheromas, with associations to major cardiovascular events.^7^ While causality remains under investigation, the precautionary principle suggests that we reduce unnecessary plastic exposure wherever possible, especially in a field that commends prevention.

Finally, the financial costs cannot be ignored. A 2009 review estimated that contact precautions increase daily care costs by over $150 per patient.^5^ In resource-constrained settings, these added costs may come at the expense of other critical interventions.

To conclude, airborne precautions remain essential for COVID-19, particularly during aerosol-generating procedures or in high-risk wards. N95 masks and eye protection should continue to be used in these contexts. However, the universal application of contact precautions, including gowns and gloves for all patients with suspected or confirmed COVID-19, should now be subjected to rigorous, scientific, pro-con debate.

While adhering to guidelines remains essential, revisiting them as science evolves is equally important. Current policies should reflect SARS-CoV-2’s transition to an endemic phase and support thoughtful, context-driven decisions. This includes balancing infection control with patient dignity, provider judgment, and environmental sustainability. Practices such as contact precautions might warrant re-evaluation as other strategies like vaccination, masking, ventilation, and respiratory hygiene, may have a more positive impact on prevention.

As health-care providers, we have a responsibility to thoughtfully assess both benefits and risks in the pursuit of evidence-based care.

## References

[1] Centers for Disease Control and Prevention. Infection control: COVID-19. https://www.cdc.gov/covid/hcp/infection-control/index.html. Published 2024. Accessed February 20, 2025.

[2] Onakpoya IJ , Heneghan CJ , Spencer EA , et al. SARS-CoV-2 and the role of fomite transmission: a systematic review. F1000Res 2021;10:233. doi: 10.12688/f1000research.51590.3 34136133 PMC8176266

[3] Rodriguez-Nava G , Diekema DJ , Salinas JL. Reconsidering the routine use of contact precautions in preventing the transmission of severe acute respiratory coronavirus virus 2 (SARS-CoV-2) in healthcare settings. Infect Control Hosp Epidemiol 2023;44:1035–1037. doi: 10.1017/ice.2023.91 37138546

[4] Rabin AS , Marr LC , Blumberg HM. Doff Thy gown—shedding contact precautions for COVID-19. Clin Infect Di 2024;79:585–587. doi: 10.1093/cid/ciae276 PMC11426274 38747695

[5] Morgan DJ , Diekema DJ , Sepkowitz K , Perencevich EN. Adverse outcomes associated with Contact Precautions: a review of the literature. Am J Infect Control 2009;37:85–93. doi: 10.1016/j.ajic.2008.04.257 19249637 PMC3557494

[6] Nihart AJ , Garcia MA , El Hayek E , et al. Bioaccumulation of microplastics in decedent human brains. Nat Med. Published online February 3, 2025. doi: 10.1038/s41591-024-03453-1 PMC12003191 39901044

[7] Marfella R , Prattichizzo F , Sardu C , et al. Microplastics and nanoplastics in atheromas and cardiovascular events. N Engl J Med 2024;390:900–910. doi: 10.1056/NEJMoa2309822 38446676 PMC11009876

